# Flying Syringes for Emerging Enzootic Virus Screening: Proof of Concept for the Development of Noninvasive Xenosurveillance Tools Based on Tsetse Flies

**DOI:** 10.1155/2023/9145289

**Published:** 2023-07-13

**Authors:** Adeline Valente, Davy Jiolle, Sophie Ravel, Angélique Porciani, Laurence Vial, Vincent Michaud, Olivier Kwiatek, Aurélie Pedarrieu, Dorothée Misse, Pauline Ferraris, François Bretagnolle, Paul-Yannick Bitome-Essono, Boris Kevin Makanga, Virginie Rougeron, Franck Prugnolle, Christophe Paupy

**Affiliations:** ^1^MIVEGEC, Montpellier University, IRD, CNRS, Montpellier, France; ^2^INTERTRYP, Montpellier University, IRD, CIRAD, Montpellier, France; ^3^ASTRE, Montpellier University, CIRAD, INRAE, Baillarguet, France; ^4^Biogéosciences, Équipe Écologie-Évolutive, CNRS-Université de Bourgogne-Franche Comté, Dijon, France; ^5^Institut de Recherche en Ecologie Tropicale, Libreville, Gabon; ^6^REHABS, International Research Laboratory, CNRS-NMU-UCBL, George Campus, Nelson Mandela University, George, South Africa

## Abstract

Pathogen transfers between wild and domestic animals and between animals and humans are increasing. Their dramatic consequences for public and veterinary health as well as for conservation call for innovative and user-friendly methods for pathogen surveillance in wildlife. Xenosurveillance, a method based on the use of invertebrates (e.g., mosquitoes, hematophagous flies, leeches, cadaveric arthropods) to sample animal tissues (e.g., blood) and the associated pathogens, is one of these tools. Previously, we demonstrated that hematophagous flies, such as tsetse flies, could be useful to detect and identify the etiological agents of malaria in a diverse range of mammals in Gabon. However, we did not assess whether this method can be also used to detect viruses. In the present study, we experimentally fed tsetse flies (*Glossina fuscipes fuscipes*) rabbit blood containing different viruses of medical or veterinary importance (Zika, Dengue, Chikungunya, African swine fever, Bluetongue, and peste des petits ruminants viruses). Then, we used quantitative PCR (i) to determine for how long viral nucleic acid fragments remained detectable in the tsetse midgut during blood digestion and (ii) to compare two blood meal preservation methods (i.e., FTA cards and RNA*later* solution) tested using tsetse flies engorged with blood and dengue-2 virus. All viruses remained detectable for 6 days after feeding, although the detection probability significantly decreased over time. FTA cards and RNA*later* solution gave similar results in terms of virus detection. Our results demonstrate that xenosurveillance using blood-engorged tsetse flies is a valuable tool to track and survey viruses in wildlife in Sub-Saharan Africa.

## 1. Introduction

Emerging and re-emerging human infectious diseases have increased in recent years mostly due to the growth of human activities that promote contacts with novel sources of pathogens and favor their spread worldwide [1]. Sixty percent of diseases emerging in humans are zoonoses, and wildlife plays a key role by providing a zoonotic pool from which previously unknown pathogens may emerge [2–5]. Many emerging threats to human health are viruses, such as human immunodeficiency virus (HIV) [6], avian influenza viruses [7], Ebola virus [8], Chikungunya virus [9, 10], Yellow fever virus [11], and more recently Zika virus [10], severe acute respiratory syndrome-related coronavirus SARS-CoV-1 and SARS-CoV-2 viruses [12] and Middle East respiratory syndrome-related coronavirus [13]. Most of these viruses circulate primarily within enzootic cycles that involve wild animals. However, occasionally, they spill over from their primary wild hosts to alternative new vertebrates, including wild and domestic animals that become sources of human infections. The recent SARS-CoV-2 crisis highlighted the urgent need to address the issue of viral emergences according to the One Health concept in order to improve the surveillance of enzootic viruses in their natural hosts before their spillover to the anthropic compartment and the occurrence of devastating epizootic or epidemic waves [14].

Besides human health, emerging viruses are also an important veterinary concern with major effects on food production stability and wild species survival. When affecting livestock, emerging viruses can cause dramatic economic losses, and threaten food security and trade. For example, the African swine fever virus (ASFV), which recently spread in China and neighboring Asian countries, caused the death or culling of more than one million pigs and seriously threatened the swine industry in Asia [15]. In addition to their spreading to livestock, some emerging viruses can propagate to wildlife with important conservation issues, especially for threatened wild species. For instance, gorillas have been infected by Ebola virus in Central Africa [16], wild ruminants by the peste des petits ruminants virus (PPRV) in Asia and Middle East [17], and lions by the canine distemper virus that decimated their populations in the Serengeti National Park in Tanzania [18].

The risk of emerging zoonotic infectious diseases is particularly elevated in tropical regions that are currently undergoing land-use changes and where the wild mammal diversity is high [19]. Surveillance and virus discovery efforts need to be directed toward mammals in these biodiversity hotspot areas. Combined with comprehensive field studies, more specific knowledge will help to refine and adapt surveillance strategies to better monitor diseases at the wildlife/human/livestock interface.

Therefore, novel and easy-to-implement surveillance strategies to detect many different pathogens that circulate among wild mammals are needed. Xenosurveillance or invertebrate DNA (iDNA), in which invertebrates are used to sample wild mammal tissues for pathogen screening, is one of these approaches. Xenosurveillance of viruses was first described by Grubaugh et al. [20] who assessed whether human viruses could be detected using mosquito blood meals. They found that HIV was detectable in the gut of mosquitoes up to 24 hr after the meal. However, their mosquito-based technique would be not suitable in wild environments where blood-engorged mosquitoes are difficult to collect [21]. Moreover, as their host preference is often restricted to a limited number of vertebrate species, different mosquito species would be needed to cover all vertebrate wildlife. Alternatively, previous studies in Gabon [22] and Tanzania [23] have shown the interest of tsetse flies to obtain the blood of the vertebrates they bite (mostly mammals, but also birds and reptiles) for the screening of bloodborne pathogens, particularly in Sub-Saharan Africa where Glossinidae are present. As, tsetse flies take large blood meals (ranging from 20 *μ*L up to 100 *μ*L for the largest species) for their activity and reproduction [24], they are useful for blood sampling without affecting wildlife. Nevertheless, it is not known whether blood meals from tsetse flies can be used to detect viruses (DNA and RNA viruses) and whether the time between blood ingestion and sample handling could affect the virus detection probability. On the other hand, these points have been already experimentally tested in other hematophagous invertebrates, for instance mosquitoes [25].

Therefore, in the present study, using an experimental approach, we assessed the relevance of using hematophagous flies as “flying syringes” for the detection/surveillance of RNA and DNA viruses of medical or veterinary interest that circulate among wild animals. Briefly, we allowed tsetse flies to feed on controlled mixtures of blood and viruses and then used molecular approaches to determine whether the virus genomes could be detected in the fly abdomens (i.e., in the blood meal) at different digestion stages. We also assessed whether two different blood meal preservation methods affected the probability of virus detection.

## 2. Materials and Methods

### 2.1. Tsetse Strain

A tsetse fly strain belonging to *Glossina fuscipes fuscipes* (colonized in the insectarium for more than 294 generations) was used for experimental infections. The strain was provided by the International Atomic Energy Agency, Insect Pest Control Laboratory, Seibersdorf, Austria as batches of pupae that were then reared to adult stages in the INTERTRYP insectarium (Baillarguet, France) in controlled environmental conditions (25°C, 80% room hygrometry, and 12 hr light: 12 hr dark cycle). Upon reception, pupae were placed in petri dishes disposed in big cages. When they emerged, tsetse flies were sorted by sex and amputated of a single wing to avoid any risk of escape. Teneral adults (2–5 days postemergence) were used for experimental infectious blood feeding.

### 2.2. Virus Selection

To assess for how long viral nucleic acids could be detected in tsetse fly guts after infectious blood meals, *G*. *f*. *fuscipes* males and females were given a blood meal that contained one of the following six emergent viruses, including five arboviruses (Table 1). They were (i) viruses of veterinary interest: ASFV (Asfaviridae/*Asfivirus*), PPRV (Paramyxoviridae/*Morbillivirus*), and Bluetongue virus (BTV; Reoviridae/*Orbivirus*) and (ii) zoonotic viruses of major concern for human health: Chikungunya virus (CHIKV; Alphaviridae/*Alphavirus*) and Dengue virus and Zika virus (DENV and ZIKV; Flaviviridae/*Flavivirus*). Their choice was guided by several considerations: (1) availability of usable viral strains, specific agreement, and infrastructure allowing their handling; (2) diversity of genome types (ssRNA/dsRNA/dsDNA); (3) viruses corresponding to several families/genera; and (4) viruses representing different modes of natural transmission. Moreover, all selected viruses are current human or veterinary health concerns and represent viruses that could be theoretically surveyed in the wild using flying syringes because they all induce viremia and can be detected in the peripheral blood of infected animals. This includes also PPRV [36, 40] although it induces a much lower viremia than arboviruses. The characteristics of viruses and strains are summarized in Table 1 and Table S1.

### 2.3. Blood feeding of Tsetse Flies

To determine the limits of pathogen detection following blood meal digestion, tsetse flies were fed with a blood/virus mixture. All blood feedings were done in BSL3 laboratories (Vectopôle of IRD, Montpellier, France for DENV, CHIKV and ZIKV; CIRAD, Baillarguet for ASFV, PPRV and BTV). For each virus, two batches containing between 40 and 90 teneral flies (one batch per sex) were fed the blood-virus mixture (2 mL of washed rabbit erythrocytes and 1 mL of viral suspension). The blood meal was supplemented with 0.5 M ATP as phagostimulant. The final viral titers in the blood meals were adapted for each tested virus by taking into account the usually reported titers in their natural hosts (Table 1). Blood/pathogen meals were provided using a Hemotek membrane feeding system. Tsetse flies were allowed to feed for 10 min through a piece of pork intestine that covered the Hemotek feeder base maintained at 37°C. After the blood meal, tsetse flies were anesthetized on ice for 5–10 min and then engorged specimens were retrieved and transferred to cardboard containers where they were maintained with 10% sucrose in controlled conditions in a climatic chamber (25 ± 1°C, 80% room hygrometry, and 12 hr light: 12 hr dark cycle) for up to 144 hr (i.e., 6 days). For each virus, experiments were replicated once (two replicates). Batches of flies were analyzed at different time points postingestion: 0, 6, 24, 48, 72, and 144 hr postingestion. For each virus and each replicate, 1–12 flies were examined at each time point.

### 2.4. Virus Detection/Quantification

Immediately after feeding (time 0) or at the different time points postfeeding, tsetse flies were killed by freezing at −80°C and dissected to remove the abdomens that were individually ground using plastic pestles in 250 *μ*L of 1x Dulbecco's phosphate-buffered saline (DPBS) solution in a 2 mL microtube. Nucleic acids were extracted from 100 *μ*L of homogenate using the NucleoSpin RNA Kit (Macherey-Nagel) and the DNeasy Blood & Tissue Kit (Qiagen) according to the manufacturers' instructions. RNA was eluted in 60 *μ*L of sterile water and stored at −80°C. DNA was eluted in 100 *µ*L of sterile water and stored at −20°C.

For RNA viruses, the detection and quantification were performed by one-step reverse transcription-quantitative polymerase chain reaction (RT-qPCR). The RT-qPCR assays were modified (i.e., by using a one-step RT-qPCR kit instead of two steps) from several previously published protocols to generate 68–209 bp amplicons located in the conserved region (see Table S2 for details). Assays were performed using the SuperScript III Platinum One-Step Quantitative Kit (Invitrogen): 1x reaction mix, 10 pmol PCR primers and probes, and 10 *µ*L RNA in a final volume of 30 *µ*L. Amplification was performed on a LightCycler 96 real-time thermocycler (Roche Diagnostics, Meylan, France) (see Table S3 for details). For ASFV (DNA virus), qPCR was performed using the SensiFast Lo-ROX Genotyping Kit (Bioline) with 1x reaction mix, 8 pmol primers, 2 pmol probe, and 2 *µ*L DNA in a final volume of 20 *µ*L. Negative controls (i.e., by replacing RNA or DNA with water instead) were used in each run. In addition we tested, a set of flies (five per time point) fed with blood without virus to search for nonspecific amplification at the different time points postfeeding.

### 2.5. Comparing FTA Cards and RNAlater for Nucleic Acid Storage

To preserve the nucleic acids present in the blood meals of tsetse flies collected in the field, the RNA*later* solution (Sigma-Aldrich) and Whatman FTA cards (WB120305, Sigma-Aldrich) were compared. Twenty-four abdomens of tsetse flies engorged with rabbit blood and DENV were isolated at 0, 6, 24, 48, 72, and 144 hr postingestion (four flies per time point), individually ground in 250 *μ*L 1X DPBS, and centrifuged at 15,000 rpm for 1 min. For each abdomen, an equal quantity of supernatant (65 *μ*L) was transferred to 100 *μ*L RNA*later* and on a 25 mm diameter FTA card sample area. RNA*later* samples were centrifuged at 15,000 rpm for 1 min, the supernatant removed, and replaced by 100 *μ*L 1X DPBS. RNA was extracted from 50 *μ*L of this solution and from half of the FTA card sample area using the NucleoSpin RNA Kit, followed by DENV qPCR analysis as described above. The efficiency of the two preservation methods was compared by calculating the positivity rates and cycle threshold (Ct) values.

### 2.6. Statistical Analyses

The linear model for each virus was adjusted to compare the Ct values between T0 and each time point after virus ingestion. The sensibility of virus detection was estimated using the brglm function with binomial distribution to estimate the error. Time points were considered as factors because they were represented by different batches of tsetse flies. The Student's *t*-test was used to determine the storage method effect on Ct. All statistical analyses were performed with the R software (version 1.2.13) [41].

## 3. Results

### 3.1. Kinetics of Virus Detection in Blood Meals

Overall, we tried to experimentally feed 1,613 tsetse flies with a blood-virus mixture. In total, 852 flies were fully engorged with the infectious mixture, and the other 759 tsetse flies did not eat and were not considered for this analysis. The engorgement rate (Table 2) ranged from 43.7% to 68.2% in function of the blood-virus mixture. The mortality rates at the different postingestion time points were comparable for the different blood-virus mixtures, except for the PPRV mixture that led to higher mortality especially at 48 hr (Table 2).

**Figure 1:**
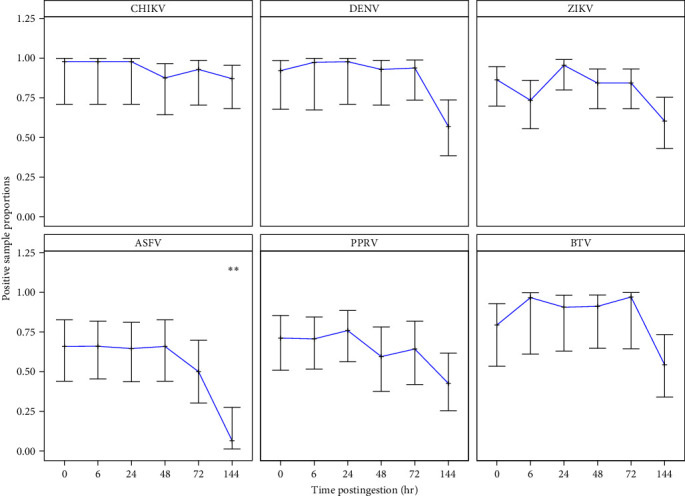
Proportion of qPCR-positive tsetse fly abdomens in function of the virus and time postingestion. CHIKV, Chikungunya virus; DENV, Dengue-2 virus; ZIKV, Zika virus; ASFV, African swine fever virus; PPRV, peste des petits ruminants virus; BTV, Bluetongue virus. Error bars: standard error calculated for three replicates. Positive sample proportions observed after 6, 24, 48, 72, and 144 hr postingestion were compared to those observed immediately after the ingestion of viruses (T0);  ^*∗∗*^*p*-value < 0.001.

**Figure 2:**
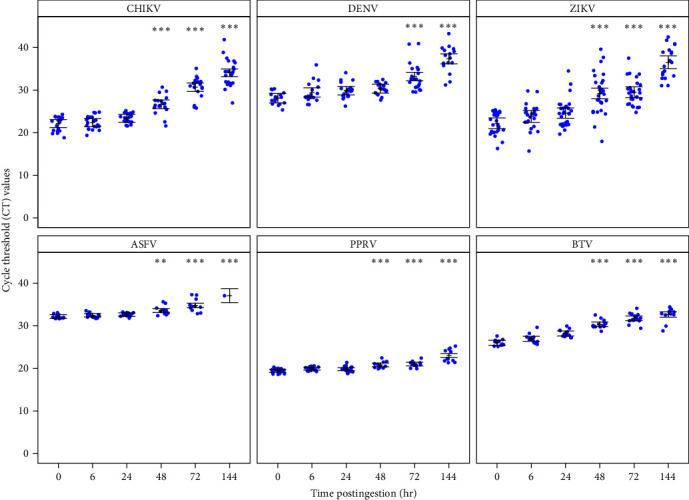
Cycle threshold (Ct) value changes over time (hours postingestion) for each virus. CHIKV, Chikungunya virus; DENV, Dengue-2 virus; ZIKV, Zika virus; ASFV, African swine fever virus; PPRV, peste des petits ruminants virus; BTV, Bluetongue virus. Error bars: standard error calculated for three replicates. Ct values observed after 6, 24, 48, 72, and 144 hr postingestion were compared to those observed immediately after the ingestion of viruses (T0);  ^*∗∗*^*p*-value < 0.001,  ^*∗∗∗*^*p*-value < 0.0001.

When analyzing tsetse flies fed with blood without virus, we detected a single nonspecific amplification (1/40) for the BTV RT-qPCR system with an associated Ct value of 42.65. We, therefore, decided not to consider positive samples with Ct values higher than 40 for this system, and we excluded from the analysis three samples that showed BTV amplifications (Ct values of 40.99, 41.11, and 42.6) after 144 hr postfeeding. The virus positivity by RT-qPCR was higher than 50% until 72 hr postingestion for all viruses, and remained high at 144 hr postingestion, except for ASFV for which the positivity rate decreased to 5% (Table 2 and Figure 1). For all tested viruses, the viral genome remained detectable in the abdomen up to 6 days (144 hr postengorgement), although the number of negative results increased significantly with time for ASFV (*p*-value_0/144 hr_ = 0.0090, Figure 1). The mean Ct value (which is inversely correlated to the target nucleic acid concentration in the sample) for each virus increased with time compared with baseline. This increase was already significant after 48 hr for all viruses (CHIKV, ZIKV, PPRV, BTV with *p*-values < 0.0001; ASFV with *p*-values = 0.0004) except DENV for which it became significant after 72 hr (*p*-values < 0.0001, Figure 2).

### 3.2. Field Storage Medium and Detection Probability

We could detect the virus in 18 and in 21 of the 24 samples of flies engorged with the blood-DENV mixture and stored on FTA cards and in the RNA*later* solution, respectively. The number of positive results (*t* = 1.2393, *df* = 25.046, *p*-value = 0.2267) and the mean Ct values (*t* = −1.1746, *df* = 35.141, *p*-value = 0.248) were not significantly different between the FTA card and RNA*later* methods.

## 4. Discussion

Our objective was to evaluate and validate the use of tsetse flies as “flying syringes” for the detection and monitoring of viruses that circulate in wildlife. Specifically, we wanted to define the temporal window of virus detection after their ingestion during a blood meal.

### 4.1. Virus Detection Capacity

Our blood-feeding experiments showed that viruses can be detected with a rather high sensitivity in blood meals of *G*. *f*. *fuscipes*. Nevertheless, the positivity rates decreased over time, from 67% to 100% just after the blood meal ingestion to 5%–88% 6 days later. This decrease in positivity rate was particularly high between day 3 and day 6 postingestion, while the rate remained roughly the same between day 0 and day 3. For all viruses (except ASFV for which the positivity rate was close to 0 after 6 days), the positivity rate remained rather high after 6 days of blood digestion (42%–88%), suggesting that tsetse flies can be effectively used to detect viruses even if the blood meal has been largely digested. For tsetse fly, the time required for a complete digestion of the blood meal content varies between 48 and 120 hr (e.g., host DNA remains detectable in the tsetse fly gut up to 7 days after blood ingestion, ravel, personal observation), while the rate of digestion has been shown to be modulated by several factors, including ambient temperature, tsetse fly activity, and experimental versus field conditions [42, 43]. Blood meal analysis of mosquitoes experimentally infected with different pathogens [44] already showed that the Ct values increase with time, probably due to the degradation of viral particles (i.e., viral genome) or of nucleic acids (DNA and RNA) during the digestion process. In mosquitoes, pathogens could be detected 24 hr after blood digestion, whereas in tsetse flies this was possible at least up to day 6 due to a longer digestion process. This indicate that using tsetse flies as “flying syringes” increases the probability of virus detection and reinforces their usefulness for virus surveillance.

These observations have practical implications for field studies. Indeed, viruses could be detected in almost all engorged tsetse flies and up to 6 days after blood feeding. This means that all collected flies are suitable for virus screening and not only those with a fresh blood meal, as previously done [22]. All abdomens can be stored (e.g., using FTA cards or RNAl*ater*, as tested here) to increase the probability of virus detection. This should largely reduce the field efforts to sort flies and to prepare and store specimens (the blood meal does not need to be isolated after dissection).

We found that the positivity rate varied among the six tested viruses. ASFV (the only DNA virus included) displayed the lowest positivity rate, with a mean detection probability of only 53.5%. Similarly, PPRV, which was the only nonarbovirus virus in our panel [45], was the second less well-detected virus (64.5%). On the other hand, the mean positivity rates for remaining arboviruses were higher with mean detection probabilities of 81.7%, 87.2%, 90.3%, and 95.3%, respectively, for ZIKV (*Flavivirus*), BTV (*Orbivirus*), DENV (*Flavivirus*), and CHIKV (*Alphavirus*). Differences in starting titers or in the molecular method sensitivity could explain the observed detection rate differences.

We noted a significant mortality of tsetse flies during experimental infection with PPRV. This may suggest a pathogenic effect related to the virus or to compounds present in the medium used for the virus culture. A previous study [46] found that blood meals containing 3% and 5% of glucose induced about 40% mortality in *Glossina morsitans submorsitans* females at 48 hr postingestion, suggesting a possible deleterious effect of sugar on tsetse fly survival. The presence of 5% of glucose in the Weybridge medium in which PPRV was lyophilized may explain the observed mortality (10.8%).

### 4.2. Choice of Blood Meal Storage Methods in the Field

Although we did not test the effect of storage temperature and time, our results suggest that the two tested storage methods (FTA cards and RNA*later*) are comparable. Nevertheless, FTA cards are more expensive than the RNA*later* solution (1.20 Euros vs. 0.07 Euros/sample). Moreover, the RNA*later* solution can even be prepared in the laboratory, for a lower cost. In addition, the use of FTA cards is impractical because it is necessary to keep part of the sample area and to extract the other part, and this require sterile cutting. From a high throughput perspective (i.e., high number of tsetse flies collected and analyzed), the use of one FTA card sample area for one fly abdomen could be restricting, whereas 10–100 specimens can be pooled together in the RNA*later* solution. Other traditional transport media also could be used to store blood meals, such as viral transport medium (VTM), at even lower cost. Regardless of the storage methods, further work using field conditions will be useful to determine the best way to maintain the sample integrity under varying environmental parameters (including storage time, temperature, and humidity).

## 5. Conclusions

Our study demonstrated that xenosurveillance methods, such as tsetse flies as flying syringes, could be used to monitor enzootic viruses that circulate in wildlife. As tsetse flies are easy to trap (little or no specific expertise required), this method will allow the simultaneous and long-term monitoring in different Sub-Saharan Africa areas. The interest of this method is increased by several studies showing that blood meals could also be used to detect circulating antibodies against specific viruses, thus broadening the information obtained from blood meals [23, 47, 48]. Overall, tsetse fly-based xenosurveillance could be very useful to monitor the circulation of enzootic viruses in the wild and at wild/human interfaces in future One Heath programs in sub-Saharan Africa.

## Figures and Tables

**Table 1:** Characteristics of the six viruses selected for tsetse fly oral infection.

Virus name (abbreviation)	Family/Genus	Presence of envelope	Genome features	Strain	Virus titer reported in vertebrate hosts	Final virus titer used for blood meals
Chikungunya (CHIKV)	*Alphaviridae*/*Alphavirus*	Yes	ssRNA(+)	LR2006_OPY1 [26]	10^1^–10^8^ PFU/mL [27]	10^6^ PFU/mL

Dengue 2 (DENV-2)	*Flaviviridae*/*Flavivirus*	Yes	ssRNA(+)	16681 [28]	10^2^–10^7^ PFU/mL [29]	10^6^ FFU/mL

Zika (ZIKV)	*Flaviviridae*/*Flavivirus*	Yes	ssRNA(+)	PF_25013-18 [30]	10^2^–10^6^ PFU/mL [31]	10^6^ PFU/mL

African swine fever (ASFV)	*Asfaviridae*/*Asfivirus*	Yes	dsDNA	LIV13/33 [32]	10^2.5^–10^3.5^ HAD_50_/mL in wild animals [33] Up to 10^8^ HAD_50_/mL in domestic animals [34]	10^3^ HAD_50_/mL

Peste des petits ruminants (PPRV)	*Paramyxoviridae/Morbillivirus*	Yes	ssRNA(−)	Maroc2008 [35]	10^6^–10^9^ RNA copies/mL in domestic animals [36]	10^7^ RNA copies/mL

Bluetongue (BTV-8)	*Reoviridae/Orbivirus*	No	dsRNA	Serotype - 8 Italy [37]	10^6^–10^8^ particles/mL in wild animals [38] 10^4^–10^7^ DL50 in domestic animals [39]	10^5.2^ TCID_50_/mL

ssRNA(+), positive-sense single-stranded RNA; ssRNA(−), negative-sense single-stranded RNA; dsRNA, double-stranded RNA; dsDNA, double-stranded DNA; PFU, plaque forming unit; FFU, focus forming unit; HAD, hemadsorbing dose; TCID, tissue culture infectious dose.

**Table 2:** Engorgement and positivity rates in tsetse flies at 0, 6, 24, 48, 72, and 144 hr postingestion of viral particles.

		Engorgement			Virus genome detection			
	Hours postingestion	No. of tsetse flies orally infected	No. of tsetse flies fully engorged	Engorgement rate (%)	No. of tsetse flies that died	No. of living tsetse flies (tested)	No. of positive tsetse flies	Positivity rate (%)
		207	128	61.8				

	0				–	21	21	100
	6				–	21	21	100
CHIKV	24				–	21	21	100
	48				–	19	17	89
	72				–	20	19	95
	144				–	26	23	88

		295	129	43.7				

	0				–	18	17	94
	6				–	18	18	100
DENV	24				–	21	21	100
	48				–	20	19	95
	72				1	23	22	96
	144				–	28	16	57

		289	197	68.2				

	0				–	32	28	88
	6				–	31	23	74
ZIKV	24				1	32	31	97
	48				–	34	29	85
	72				–	34	29	85
	144				–	33	20	61

		307	135	44				

	0				–	21	14	67
	6				–	24	16	67
ASFV	24				–	23	15	65
	48				–	21	14	67
	72				–	22	11	50
	144				2	22	1	5

		317	166	52.4				

	0				–	25	18	72
	6				–	28	20	71
PPRV	24				1	26	20	77
	48				16	20	12	60
	72				4	20	13	65
	144				–	26	11	42

		198	99	50				

	0				–	16	13	81
	6				–	14	14	100
BTV	24				–	15	14	93
	48				–	16	15	94
	72				–	16	16	100
	144				–	22	12	55

CHIKV, Chikungunya virus; DENV, Dengue-2 virus; ZIKV, Zika virus; ASFV, African swine fever virus; PPRV, Peste des petits ruminants virus; BTV, bluetongue virus.

## Data Availability

The databases related to positivity rates/Ct values and scripts used for statistical analyses with R to support the findings of this study are available from the corresponding author upon request.

## Supplementary Materials

Supplementary Materials Table S1: details on virus culture methods used for CHIKV, DENV, ZIKV, ASFV, PPRV, and BTV. Table S2: details on QPCR systems used for the detection of CHIKV, DENV, ZIKV, ASFV, PPRV, and BTV genomes in tsetse fly abdomens. Table S3: cycling conditions used for QPCR targeting CHIKV, DENV, ZIKV, ASFV, PPRV, and BTV.

## References

[1] Murray K. A., Preston N., Allen T., Zambrana-Torrelio C., Hosseini P. R., Daszak P. (2015). Global biogeography of human infectious diseases. *Proceedings of the National Academy of Sciences*.

[2] Taylor L. H., Latham S. M., Woolhouse M. E. (2001). Risk factors for human disease emergence. *Philosophical Transactions of the Royal Society of London. Series B, Biological Sciences*.

[3] Daszak P., Tabor G. M., Kilpatrick A. M., Epstein J., Plowright R. (2004). Conservation medicine and a new agenda for emerging diseases. *Annals of the New York Academy of Sciences*.

[4] Woolhouse M., Gaunt E. (2007). Ecological origins of novel human pathogens. *Critical Reviews in Microbiology*.

[5] Jones K. E., Patel N. G., Levy M. A. (2008). Global trends in emerging infectious diseases. *Nature*.

[6] Sharp P. M., Hahn B. H. (2011). Origins of HIV and the AIDS pandemic. *Cold Spring Harbor Perspectives in Medicine*.

[7] Alexander D. J. (2007). An overview of the epidemiology of avian influenza. *Vaccine*.

[8] Baize S., Pannetier D., Oestereich L. (2014). Emergence of Zaire Ebola virus disease in Guinea. *New England Journal of Medicine*.

[9] Burt F. J., Rolph M. S., Rulli N. E., Mahalingam S., Heise M. T. (2012). Chikungunya: a re-emerging virus. *The Lancet*.

[10] Wikan N., Smith D. R. (2016). Zika virus: history of a newly emerging arbovirus. *The Lancet Infectious Diseases*.

[11] Paules C. I., Fauci A. S. (2017). Yellow fever—once again on the radar screen in the Americas. *New England Journal of Medicine*.

[12] Zhou P., Yang X.-L., Wang X.-G. (2020). A pneumonia outbreak associated with a new coronavirus of probable bat origin. *Nature*.

[13] de Wit E., van Doremalen N., Falzarano D., Munster V. J. (2016). SARS and MERS: recent insights into emerging coronaviruses. *Nature Reviews Microbiology*.

[14] Cunningham A. A., Daszak P., Wood J. L. N. (2017). One health, emerging infectious diseases and wildlife: two decades of progress?. *Philosophical Transactions of the Royal Society B: Biological Sciences*.

[15] Normile D. (2019). African swine fever marches across much of Asia. *Science*.

[16] Bermejo M., Rodríguez-Teijeiro J. D., Illera G., Barroso A., Vilà C., Walsh P. D. (2006). Ebola outbreak killed 5000 gorillas. *Science*.

[17] Aguilar X. F., Fine A. E., Pruvot M. (2018). PPR virus threatens wildlife conservation. *Science*.

[18] Viana M., Cleaveland S., Matthiopoulos J. (2015). Dynamics of a morbillivirus at the domestic–wildlife interface: canine distemper virus in domestic dogs and lions. *Proceedings of the National Academy of Sciences of the United States of America*.

[19] Allen T., Murray K. A., Zambrana-Torrelio C. (2017). Global hotspots and correlates of emerging zoonotic diseases. *Nature Communications*.

[20] Grubaugh N. D., Sharma S., Krajacich B. J. (2015). Xenosurveillance: a novel mosquito-based approach for examining the human-pathogen landscape. *PLOS Neglected Tropical Diseases*.

[21] Makanga B., Costantini C., Rahola N. (2017). Show me which parasites you carry and I will tell you what you eat”, or how to infer the trophic behavior of hematophagous arthropods feeding on wildlife. *Ecology and Evolution*.

[22] Bitome-Essono P.-Y., Ollomo B., Arnathau C. (2017). Tracking zoonotic pathogens using blood-sucking flies as ‘flying syringes’. *eLife*.

[23] Mwakasungula S., Rougeron V., Arnathau C. (2022). Using haematophagous fly blood meals to study the diversity of blood-borne pathogens infecting wild mammals. *Molecular Ecology Resources*.

[24] Laveissière C., Grébaut P., Herder S., Penchenier L. (2000). *Les glossines vectrices de la maladie du sommeil*.

[25] Yang Y., Garver L. S., Bingham K. M. (2015). Feasibility of using the mosquito blood meal for rapid and efficient human and animal virus surveillance and discovery. *The American Journal of Tropical Medicine and Hygiene*.

[26] Ekchariyawat P., Hamel R., Bernard E. (2015). Inflammasome signaling pathways exert antiviral effect against Chikungunya virus in human dermal fibroblasts. *Infection, Genetics and Evolution*.

[27] Appassakij H., Khuntikij P., Kemapunmanus M., Wutthanarungsan R., Silpapojakul K. (2013). Viremic profiles in asymptomatic and symptomatic chikungunya fever: a blood transfusion threat?. *Transfusion*.

[28] Luplertlop N., Surasombatpattana P., Patramool S. (2011). Induction of a peptide with activity against a broad spectrum of pathogens in the *Aedes aegypti* salivary gland, following infection with dengue virus. *PLoS Pathogens*.

[29] Yamada K. I., Takasaki T., Nawa M., Kurane I. (2002). Virus isolation as one of the diagnostic methods for dengue virus infection. *Journal of Clinical Virology*.

[30] Hamel R., Dejarnac O., Wichit S. (2015). Biology of Zika Virus infection in human skin cells. *Journal of Virology*.

[31] Faye O., Faye O., Diallo D., Diallo M., Weidmann M., Sall A. A. (2013). Quantitative real-time PCR detection of Zika virus and evaluation with field-caught mosquitoes. *Virology Journal*.

[32] Rennie L., Wilkinson P. J., Mellor P. S. (2000). Effects of infection of the tick *Ornithodoros moubata* with African swine fever virus. *Medical and Veterinary Entomology*.

[33] Thomson G. R., Gainaru M. D., Van Dellen A. F. (1980). Experimental infection of warthos (Phacochoerus aethiopicus) with African swine fever virus. *The Onderstepoort Journal of Veterinary Research*.

[34] Guinat C., Reis A. L., Netherton C. L., Goatley L., Pfeiffer D. U., Dixon L. (2014). Dynamics of African swine fever virus shedding and excretion in domestic pigs infected by intramuscular inoculation and contact transmission. *Veterinary Research*.

[35] Hammouchi M., Loutfi C., Sebbar G. (2012). Experimental infection of alpine goats with a moroccan strain of peste des petits ruminants virus (PPRV). *Veterinary Microbiology*.

[36] Enchery F., Hamers C., Kwiatek O. (2019). Development of a PPRV challenge model in goats and its use to assess the efficacy of a PPR vaccine. *Vaccine*.

[37] Di Gialleonardo L., Migliaccio P., Teodori L., Savini G. (2011). The length of BTV-8 viraemia in cattle according to infection doses and diagnostic techniques. *Research in Veterinary Science*.

[38] Verwoerd D. W., Erasmus B. J., Cioetzer J. A. W., Thomson G. R., Tustin R. C. (1994). *Bluetongue. Infectious Disease of Livestock with Special Reference to Southern Africa*.

[39] Drolet B. S., Reister L. M., Rigg T. D. (2013). Experimental infection of white-tailed deer (*Odocoileus virginianus*) with Northern European bluetongue virus serotype 8. *Veterinary Microbiology*.

[40] Michaud V., Gil P., Kwiatek O. (2007). Long-term storage at tropical temperature of dried-blood filter papers for detection and genotyping of RNA and DNA viruses by direct PCR. *Journal of Virological Methods*.

[41] R Core Team (2021). *R: A Language and Environment for Statistical Computing*.

[42] Leak S. G. A. (1998). *Tsetse Biology and Ecology: Their Role in the Epidemiology and Control of Trypanosomosis*.

[43] McCue M. D., Boardman L., Clusella-Trullas S., Kleynhans E., Terblanche J. S. (2016). The speed and metabolic cost of digesting a blood meal depends on temperature in a major disease vector. *Journal of Experimental Biology*.

[44] Fauver J. R., Gendernalik A., Weger-Lucarelli J. (2017). The use of xenosurveillance to detect human bacteria, parasites, and viruses in mosquito bloodmeals. *The American Journal of Tropical Medicine and Hygiene*.

[45] EFSA AHAW Panel (EFSA Panel on Animal Health and Welfare) (2015). Scientific opinion on peste des petits ruminants. *EFSA Journal*.

[46] Solano P., Salou E., Rayaisse J.-B. (2015). Do tsetse flies only feed on blood?. *Infection, Genetics and Evolution*.

[47] Barbazan P., Palabodeewat S., Nitatpattana N., Gonzalez J.-P. (2009). Detection of host virus-reactive antibodies in blood meals of naturally engorged mosquitoes. *Vector-Borne and Zoonotic Diseases*.

[48] Štefanić S., Grimm F., Mathis A., Winiger R., Verhulst N. O. (2022). Xenosurveillance proof-of-principle: detection of *Toxoplasma gondii* and SARS-CoV-2 antibodies in mosquito blood meals by (pan)-specific ELISAs. *Current Research in Parasitology & Vector-Borne Diseases*.

