# Supplementary material for: Flying Syringes for Emerging Enzootic Virus Screening: Proof of Concept for the Development of Noninvasive Xenosurveillance Tools Based on Tsetse Flies
Source: Transbound Emerg Dis. 2023 Jul 13;2023:9145289. doi: 10.1155/2023/9145289 (PMC12016974; doi:10.1155/2023/9145289)
Supplement: Supplementary Materials — Table S1: details on virus culture methods used for CHIKV, DENV, ZIKV, ASFV, PPRV, and BTV. Table S2: details on QPCR systems used for the detection of CHIKV, DENV, ZIKV, ASFV, PPRV, and BTV genomes in tsetse fly abdomens. Table S3: cycling conditions used for QPCR targeting CHIKV, DENV, ZIKV, ASFV, PPRV, and BTV. [file 9145289.f1.docx]

**Supplemental Table 1 :**

| **Virus** | **Propagation method** | **Titration method** | **Reference for titration method** |
| --- | --- | --- | --- |
| **CHIKV** | On C6/36 cells with DMEM supplemented with 0.1% penicillin (10,000 U/ml)/streptomycin (10,000 µg/ml), 2% of decomplemented fetal bovine serum (FBS) and 1x non-essential amino acids | Plaque Assay | Jiolle, D, Moltini-Conclois, I, Obame-Nkoghe, J, Yangari, P, Porciani, A, Scheid, B, Kengne, P, Ayala D, Failloux, AB, Paupy, C. (2021). Experimental infections with Zika virus strains reveal high vector competence of *Aedes albopictus*and *Aedes aegypti* populations from Gabon (Central Africa) for the African virus lineage. *Emerg Microbes Infec*t*.* 10(1):1244-1253. doi: 10.1080/22221751.2021.1939167. |
| **ZIKV** |  |  |  |
| **DENV** |  | Fluorescent Focus Assay | Fontaine, A, Jiolle, D, Moltini-Conclois, I, Lequime, S, Lambrechts, L. (2016) Excretion of dengue virus RNA by Aedes aegypti allows non-destructive monitoring of viral dissemination in individual mosquitoes. *Sci Rep*. 6:24885. doi: 10.1038/srep24885. |
| **ASFV** | On porcine alveolar macrophages with MEM with Earle's salts supplemented with 0.2% penicillin (10,000 U/ml)/streptomycin (10,000 µg/ml) and 0.5 µg/mL amhotericin B | Hemadsorption | Carrascosa, AL, Santarén, JF, Viñuela, E. (1982). Production and titration of African swine fever virus in porcine alveolar macrophages. *J Virol Methods.* 3(6):303-10. doi: 10.1016/0166-0934(82)90034-9. |
| **PPRV** | On Vero cells with MEM supplemented with 5% of FBS, then freeze-dried in Weybridge medium | RNA copies/mL (qPCR) | Enchery, F, Hamers, C, Kwiatek, O, Gaillardet, D, Montange, C, Brunel, H, Goutebroze, S, Philippe-Reversat, C, Libeau, G, Hudelet, P, Bataille, A. (2019). Development of a PPRV challenge model in goats and its use to assess the efficacy of a PPR vaccine. *Vaccine*. 37(12):1667-1673. doi: 10.1016/j.vaccine.2019.01.057. |
| **BTV** | On Vero cells with MEM supplemented with 5% of FBS | Tissue Culture Infectious Dose 50%/mL | Reed LJ, Muench H. (1938). A simple method of estimating fifty percent endpoints. *Am J Epidemiol*. 27:493–497. https://doi.org/10.1093/oxfordjournals.aje.a118408 |

**Supplemental Table 2 :**

| **Virus** | **Gene** | **Primers/Probe** | **Sequence 5’ - 3’** | **Amplicon length (bp)** | **Reference** |
| --- | --- | --- | --- | --- | --- |
| **CHIKV** | E1, structural protein | R-CHIK | CCAAATTGTCCYGGTCTTCCT | 209 | Pastorino, B, Bessaud, M, Grandadam, M, Murri, S, Tolou, H. J., & Peyrefitte C. N. (2005). Development of a TaqMan RT-PCR assay without RNA extraction step for the detection and quantification of African Chikungunya viruses. *J Virol Methods124*(1-2):65-71. doi: 10.1016/j.jviromet.2004.11.002. |
|  |  | F-CHIK | AAGCTYCGCGTCCTTTACCAAG |  |  |
|  |  | P-CHIK | FAM-CCAATGTCYTCMGCCTGGACACCTTT-TAMRA |  |  |
| **DENV** | 3’UTR, conserved gene | DR | CGYTCTGTGCCTGGAWTGAT | 108 | Leparc-Goffart, I., Baragatti, M., Temmam, S., Tuiskunen, A., Moureau, G., Charrel, R., & de Lamballerie X. (2009). Development and validation of real-time one-step reverse transcription-PCR for the detection and typing of dengue viruses. *J Clin Virol. 45*(1):61-6. doi: 10.1016/j.jcv.2009.02.010. |
|  |  | DF | AGGACYAGAGGTTAGAGGAGA |  |  |
|  |  | 1-4DP | FAM-ACAGCATATTGACGCTGGGARAGACC-TAMRA |  |  |
| **ZIKV** | NS1, non-structural protein | ZIKA2-S | CTTGGAGTGCTTGTGATT | 187 | Atieh, T., Baronti, C., de Lamballerie, X., & Nougairède A. (2016). Simple reverse genetics systems for Asian and African Zika viruses. *Sci Rep. 2016 6*:39384. doi: 10.1038/srep39384. |
|  |  | ZIKA2-R | CTCCTCCAGTGTTCATTT |  |  |
|  |  | ZIKA223 P | FAM-AAGAAGAGAATGACCACAAAGATCATC-TAMRA |  |  |
| **ASFV** | VP72, envelope protein | ASF–VP72-F | CCCAGGRGATAAAATGACTG | 68 | Fernández-Pinero, J., Gallardo, C., Elizalde, M., Robles, A., Gómez, C., Bishop, R., . . . Arias M. (2013). Molecular diagnosis of African Swine Fever by a new real-time PCR using universal probe library. *Transbound Emerg Dis. 60*(1):48-58. doi: 10.1111/j.1865-1682.2012.01317.x. |
|  |  | ASF-VP72-R | CACTRGTTCCCTCCACCGATA |  |  |
|  |  | UPL#162 | FAM-GGCCAGGA-Dark quencher |  |  |
| **PPRV** | N protein, nucleocapsid | NPPRf | GAGTCTAGTCAAAACCCTCGTGAG | 97 | Kwiatek, O., Keita, D., Gil, P., Fernández-Pinero, J., Jimenez Clavero, M. A., Albina, E., & Libeau, G. (2010). Quantitative one-step real-time RT-PCR for the fast detection of the four genotypes of PPRV. *J Virol Methods 165*(2):168-77. doi: 10.1016/j.jviromet.2010.01.014. |
|  |  | NPPRr | TCTCCCTCCTCCTGGTCCTC |  |  |
|  |  | NPPRp | FAM-CGGCTGAGGCACTCTTCAGGCTGC-BHQ1 |  |  |
| **BTV** | NS1, non-structural protein | BTV_S5_F | GGCAACYACCAAACATGGA | 76 | Toussaint, J. F., Sailleau, C., Breard, E., Zientara, S., & De Clercq, K. (2007). Bluetongue virus detection by two real-time RT-qPCRs targeting two different genomic segments. *J Virol Methods. 140*(1-2):115-23. doi: 10.1016/j.jviromet.2006.11.007. |
|  |  | BTV_S5_R | AAAGTYCTCGTGGCATTWGC |  |  |
|  |  | BTV_S5_P | FAM-CYCCACTGATRTTGATTTTCTCAA-BHQ1 |  |  |

**Supplemental Table 3 :**

| **Virus** | **Reverse transcription** | **Initial denaturation** | **Number of cycles** | **Denaturation** | **Annealing/Elongation** |
| --- | --- | --- | --- | --- | --- |
|  |  |  |  |  |  |
| **CHIKV** | 50°C for 30 min | 95°C for 2 min | 45 | 95°C for 15 sec | 60°C for 45 sec |
|  |  |  |  |  |  |
| **DENV** |  |  |  |  |  |
|  |  |  |  |  |  |
| **ZIKV** |  |  |  |  |  |
|  |  |  |  |  |  |
| **PPRV** |  |  |  |  |  |
|  |  |  |  |  |  |
| **BTV** |  |  |  |  | 50°C for 45 sec |
|  |  |  |  |  |  |
| **ASFV** | NA | 95°C for 5 min |  | 95°C for 10 sec | 60°C for 30 sec |
|  |  |  |  |  |  |
